# Mechanic’s Hand Heralding Relapse in an Indian Adolescent with Anti-MDA5 Positive Juvenile Dermatomyositis

**DOI:** 10.31138/mjr.33.2.268

**Published:** 2022-06-30

**Authors:** Keya Ganatra, Rohit Aggarwal, Latika Gupta

**Affiliations:** 1 Seth Gordhandas Sunderdas Medical College & King Edward Memorial Hospital, Mumbai, India,; 2 Department of Medicine, University of Pittsburgh, Pittsburgh, PA, United States,; 3 Department of Clinical Immunology and Rheumatology, Sanjay Gandhi Postgraduate Institute of Medical Sciences, Lucknow, India

**Keywords:** autoimmune myositis, juvenile dermatomyositis, anti-MDA5 antibodies

## Abstract

Anti-MDA5 antibodies characterise a distinct phenotype of dermatomyositis in adults as well as children, with ethnic disparity in clinical presentation and severity. They often present as a diagnostic conundrum with rash, ulceration, and polyarthritis, but minimal muscle disease. Mechanic’s hands are typically associated with anti-synthetase syndrome, but their presence in anti-MDA5 antibody positive patients, although reported, is not well known. We present the case of a boy in whom mechanic’s hand heralded a relapse of juvenile dermatomyositis which was suspected based on remotely assessed patient-reported outcome measures on teleconsultation. This report suggests that mechanic’s hands should also prompt testing for myositis antibodies including anti-MDA5 in Indian children with JDM. Diligent awareness of the condition, and timely use of patient reported outcome measures of muscle power and skin assessment may guide management while delivering remote care in challenging situations such as a global pandemic.

Dear Editor,

An 18-year-old boy diagnosed with juvenile dermatomyositis (JDM) three years ago in remission on Methotrexate presented with erythematous rashes after disrupted medication supply due to logistic challenges during the COVID-19 pandemic. A relapse was suspected on Teleconsultation with a two-minute walk distance of 147.9 metres (normal >180 metres) and ten times arm lift time of 12.02 seconds (normal ∼10–12 seconds). Physical examination confirmed relapse, with initial development of mechanic’s hands, followed a week later by a periorbital Heliotrope rash, Gottron’s papules, patchy alopecia, and hyperpigmentation of ear lobes in areas of previous healed rashes (figure 1A–F). Manual muscle testing 8 score was 61 (maximum of 80) and muscle enzymes elevated (CK 213 U/I, LDH 1013 U/I, AST 140 U/I, ALT 81 U/I). The patient had no dyspnea, shortness of breath or other respiratory symptoms. On initial presentation 3 years ago, he was diagnosed with anti-Melanoma Differentiation Associated protein (MDA5) antibodies associated JDM when he presented with classic JDM rashes, muscle weakness, and bilateral basal ground glass opacities on contrast enhanced CT of chest suggestive of interstitial lung disease (ILD). However, the patient had no rash indicative of mechanic’s hands at the time of initial presentation.

**Figures:**
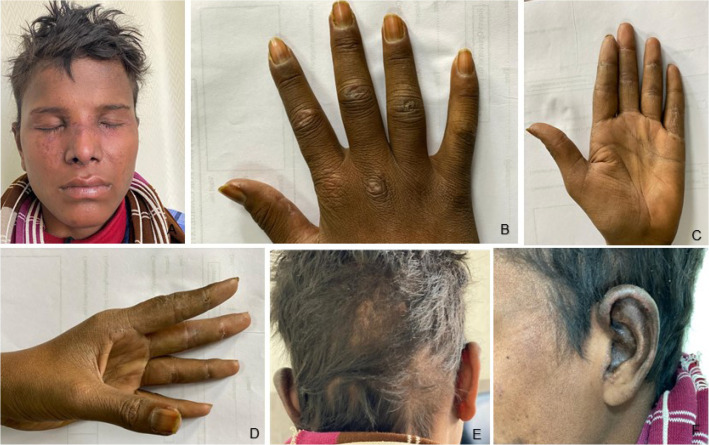
(**A**) Heliotrope Rash and lip oedema (**B**) Gottron’s papules (**C, D**) Mechanic’s hand (**E**) Patchy alopecia (**F**) Hyperpigmentation of ear lobes.

Anti-MDA5 antibodies characterise a distinct phenotype in adults as well as children, with ethnic disparity in clinical presentation and severity.^1^ They often present as a diagnostic conundrum with rash, ulceration, and polyarthritis but minimal muscle disease. Severe forms of ILD are reportedly limited to the Japanese population. This case suggests that a similar phenotype may be manifest in Indian adolescents with JDM in association with chronic ILD.^3^ Mechanic’s hands are typically associated with anti-synthetase syndrome but its presence in anti-MDA5 antibody positive patients, although reported, is not well known.^2,3^ Thus, mechanic’s hand should also prompt testing for myositis antibodies including anti-MDA5 in Indian children with JDM. Diligent awareness of the condition, and timely use of remote measures of muscle power and skin assessment may guide management while delivering remote care in challenging situations, such as a global pandemic.^4^

## KEY FEATURES:

1. The presence of Mechanic’s hands in anti-MDA5 antibody positive patients, although reported, is not well known.
2. This report suggests that mechanic’s hands should also prompt testing for myositis antibodies including anti-MDA5 in Indian children with JDM.

## References

[1] Tansley SL Betteridge ZE Gunawardena H Jacques TS Owens CM Pilkington C Anti-MDA5 autoantibodies in juvenile dermatomyositis identify a distinct clinical phenotype: a prospective cohort study. Arthritis Res Ther 2014;16:R138. 24989778 10.1186/ar4600 PMC4227127

[2] Allenbach Y Uzunhan Y Toquet S Leroux G Gallay L Marquet A Different phenotypes in dermatomyositis associated with anti-MDA5 antibody: Study of 121 cases. Neurology 2020 J;95:e70–8. 32487712 10.1212/WNL.0000000000009727 PMC7371381

[3] Gupta L Naveen R Gaur P Agarwal V Aggarwal R. Myositis-specific and myositis-associated autoantibodies in a large Indian cohort of inflammatory myositis. Semin Arthritis Rheum 2021;51:113–20. 33360322 10.1016/j.semarthrit.2020.10.014

[4] Gupta L Lilleker JB Agarwal V Chinoy H Aggarwal R. COVID-19 and myositis – unique challenges for patients. Rheumatology 2021;60:907–10. 33175137 10.1093/rheumatology/keaa610 PMC7717379

